# Novel patterning of nano-bioceramics: template-assisted electrohydrodynamic atomization spraying

**DOI:** 10.1098/rsif.2007.1162

**Published:** 2007-08-16

**Authors:** X Li, J Huang, M.J Edirisinghe

**Affiliations:** Department of Mechanical Engineering, University College LondonTorrington Place, London WC1E 7JE, UK

**Keywords:** topography, hydroxyapatite, nanoparticles, template-assisted, electrohydrodynamic, spraying

## Abstract

The ability to create patterns of bioactive nanomaterials particularly on metallic and other types of implant surfaces is a crucial feature in influencing cell response, adhesion and growth. In this report, we uncover and elucidate a novel method that allows the easy deposition of a wide variety of predetermined topographical geometries of nanoparticles of a bioactive material on both metallic and non-metallic surfaces. Using different mesh sizes and geometries of a gold template, hydroxyapatite nanoparticles suspended in ethanol have been electrohydrodynamically sprayed on titanium and glass substrates under carefully designed electric field conditions. Thus, different topographies, e.g. hexagonal, line and square, from hydroxyapatite nanoparticles were created on these substrates. The thickness of the topography can be controlled by varying the spraying time.

## 1. Introduction

Hydroxyapatite (HA) is well known to promote direct bone apposition and therefore osteoconductive. Nanometre-scale apatite crystallites form the inorganic constituent of human bone (Currey 1983) and therefore the quest to deposit such crystals on an actual implant surface, e.g. titanium, has generated significant interest in past years (Racey *et al*. 2006). Metallic implants with uniform HA coating have been used very successfully in dental and orthopaedic applications (Wolke *et al*. 1994; Takaoka *et al*. 1996; De Groot *et al*. 1998).

Recently, attention has focused on creating a well-defined surface topography of biomaterials, a feature that is known to affect initial biological response (Curtis & Wilkinson 1997). A number of studies have been conducted to evaluate the relationship between surface structure, dimensions and tissue growth on HA (Rosa *et al*. 2003; Tan & Saltzman 2004). These findings suggest that cell activity could be upregulated through optimization of the surface properties of the substrates. Thus, to fulfil this need, the development of a second generation coating procedure, which allows easy, selective and well-defined patterning, is required.

Electrohydrodynamic processes basically offer the capability of producing either uniform coating by spraying or patterning by printing (Leeuwenburgh *et al*. 2002; Ahmad *et al*. 2006). In terms of biological response, printing patterns with topography can be much more effective than simply depositing a continuous coating on the substrate (Huang *et al*. 2004*a*,*b*). Coating by spraying alone is incapable of depositing patterns, and printing is not very effective on metallic surfaces and the print-patterning of micrometre-scale islands such as those achieved in this work is difficult. There has been no attempt to couple coating and printing with templating. Basically, the electrohydrodynamic process consists of a ground electrode and a nozzle (needle) connected to a high-voltage supply. A suspension or liquid containing the material to be deposited is fed to the nozzle by a pump at a controlled flow rate. The surface of flowing medium is subjected to an electric field generated by the applied voltage between the nozzle and ground electrode, and deforms locally to an elongated jet which subsequently detaches from the body of the liquid and disintegrates into droplets. The jet and droplet generations are classified into different modes of spraying by the geometries of the jetting (Jaworek & Krupa 1999). Out of several different modes, the cone-jet mode, which is the steadiest mode of spraying, could regularize the break-up of the jet to generate fine and uniform droplets of a few micrometres in size. The process also offers the attractive advantages of easy control of a large uniform area coverage, low cost, compatibility with micro-fabrication technology and suitability for industrial production.

In this work, well-defined nano-HA micro-scale patterning on conducting and non-conducting surfaces by template-assisted electrohydrodynamic atomization (TAEA) spraying is uncovered and elucidated. Various templates having different mesh sizes and geometries were used to regulate the topography of nHA deposited, and the spraying time was used to control the thickness of the deposit.

## 2. Materials and methods

### 2.1 Preparation of suspension

Nano-sized HA (nHA) was synthesized based on a wet precipitation reaction between calcium hydroxide and orthophosphoric acid, both obtained from BDH, UK. To 0.5 M calcium hydroxide solution, 0.3 M orthophosphoric acid was added dropwise under continuous stirring at room temperature, while the pH was kept above 10.5 by the addition of ammonia solution. The precipitates obtained were further aged for two weeks and then washed with boiling water. The aged nHA particles were taken up in ethanol to prepare a 6 wt% suspension suitable for the electrohydrodynamic flow process. Further details of suspension preparation and characterization of the nHA particles and suspension are described elsewhere (Huang *et al*. 2004*a*,*b*).

### 2.2 Characterization of suspension

The nHA particles dispersed in ethanol were collected on grids with carbon supporting film. Their morphology was examined under a JEOL 200CX transmission electron microscope (TEM) operated at 200 keV. The structure of the nHA was analysed using a Phillip X-ray diffractometer operating at 40 kV and 40 mA in the scanning range of 25°–50° with a step size of 0.05° and a scan time of 6 s. The density, pH, surface tension, viscosity, electrical conductivity and relative permittivity of nHA suspension were measured as these properties affect the electrohydrodynamic flow processing. The densities of the samples were calculated using the standard density bottle. Surface tension was measured using a Kruss Tensiometer K9 (Du Novy's ring method). Viscosity was evaluated using a Visco-Easy rotational viscometer. Electrical conductivity was assessed using a HACH SensION 156 probe. Relative permittivity was estimated using the standard capacitance method. All the equipments used were initially calibrated using ethanol to validate reference data.

### 2.3 Template-assisted spray deposition

The general equipment layout used in this work is illustrated in figure 1*a*. The stainless steel needle used had an inner diameter of approximately 300 μm and two ground electrode–substrate configurations were used. One was a directly grounded substrate (figure 1*b*) and the other was the substrate kept on a silicon wafer fully covering a metallic ground electrode (figure 1*c*). In both cases, the template, which determined the architecture of the pattern deposited, was placed on the substrate. Gold templates of different mesh sizes and geometries were used in the experiments.

Freshly prepared 6 wt% nHA suspension was syringed to the needle at flow rates of 1–35 μl min^−1^ with the applied voltage between the needle and the ground electrode varying up to 6 kV to investigate electrohydrodynamic spraying scenarios. The distance between the stainless steel needle and substrate (*D*) was fixed at 20 mm. Standard silica glass microscope slides and titanium plates, the latter polished using P4000 silicon carbide grinding paper, were used as substrates to be deposited with nHA. The thickness of glass microscope slides and titanium plates are 1 and 2 mm, respectively.

### 2.4 Characterization of deposits

The morphology and thickness of the nano-HA deposits on substrates were examined using a JEOL 6301 field emission scanning electron microscope. The relationship between the nHA patterning thickness and spray time was determined by studying the cross section of the deposit. The thickness at a specific spray time was evaluated by taking the mean thickness of three coated substrates.

## 3. Results and discussion

### 3.1 Suspension characteristics

X-ray diffraction results (figure 2*a*) revealed the presence of all the major HA peaks, and no secondary phase was detected, which confirmed the purity of nHA prepared. TEM examination (figure 2*b*) revealed that the nHA particles were 75±23 nm long and 39±7 nm wide.

The properties of the nHA suspension are compared with those of ethanol. Ethanol is well known to give stable cone-jet mode electrohydrodynamic jetting and droplet generation (Grace & Marijnissen 1994), and its surface tension, electrical conduction and relative permittivity are key parameters in achieving this. The nHA particles make the ethanol more dielectric reducing the electrical conductivity from 3.4×10^−4^ to 1×10^−4^ S m^−1^ and increasing the relative permittivity from 26 to 76. However, the most significant change is in the viscosity that has increased by five times to 6.8 mPa s and this will give rise to coarser droplets (Jayasinghe & Edirisinghe 2002). The density and surface tension of nHA suspension are 930 kg m^−3^ and 28 mN m^−1^, respectively.

### 3.2 Patterning process

Two key process control parameters, namely applied voltage and flow rate, are crucial to achieve the stable cone-jet mode. The relationship between applied voltage and flow rate for this nHA suspension is shown in figure 3*a* and this map shows that the cone-jet mode can be achieved only when the flow rate and the applied voltage are in a distinctive envelope. In this work, an applied voltage range of 4.3–5.2 kV and a flow rate of 20 μl min^−1^ were used and stable cone-jetting (figure 3*b*) was maintained throughout topography generation. When the spray time was 15 s, the thickness of the nHA deposit is approximately 1.4 μm. Along with the increase of the spraying time, the thickness of the deposit was found to increase to 4.9±0.5 μm at 120 s. Therefore, as expected, the deposit thickness is directly proportional to the spraying time.

### 3.3 Topography generation

When sprayed using the electrode configuration shown in figure 1*b*, most of the suspension droplets attached onto the gold template rather than the substrate, and nHA topographies cannot be generated on the substrate in this way. In comparison, the set-up shown in figure 1*c* was much more amenable and efficient in the generation of a predetermined topography since the substrate and template are not grounded. Between the ground electrode and substrate, there is silicon wafer (approx. 1 mm) which is dielectric. During spraying, droplets are driven by the electric field generated between the needle and the grounded conducting metal plate. Therefore, in the relatively uniform electric field, the droplets could move and attach onto the template and substrate uniformly, which is a significant factor in creating topographies on the substrate as the template is just simply removed after spraying.

The droplet relics adhere well to the metal substrate. This is to be expected as the charged suspension droplets discharge particularly on the metallic surface. The deposits are usually heat treated to enhance adhesion to the substrate and our preliminary work on this is reported and discussed elsewhere (Li *et al*. in press).

As shown in figure 4*a*, by using a hexagonal template, HA nanoparticles distributed into hexagonal islands on the glass surface can be prepared in this way. The diameters of the islands could be controlled at 50 μm and the spaces between the islands are 10 μm. By using parallel and square templates on a polished titanium substrate, this method also enabled the creation of line and square topography (figure 4*b*,*c*) with each line of approximately 50 μm width and the distance between the lines was approximately 80 μm, and each square of side approximately 50 μm and the space between the square islands was approximately 20 μm. Figure 4*b* exhibits the nanostructured particle assembly in the deposits. Nano-scale HA more closely mimics HA crystals in bone and thereby enhances bioactivity (Lewandrowski *et al*. 2003; Wang *et al*. 2004).

All nHA patterns prepared in this way exhibited uniform and ordered topography. The spaces between the islands were kept constant and varied only by a few micrometres, and by using the same template, the shape and size of islands in the entire coating were kept the same. Since the spray time was controlled at 30 s during the process, the height of the ‘islands’ in the coatings was approximately 2 μm. The line patterns in figure 4*c* can be compared directly with those generated using the electrohydrodynamic printing work (Ahmad *et al*. 2006) and indeed the resolution, scatter control and uniformity of the patterns deposited in this work are superior.

The template can also be used as a metal substrate as it is coated according to the ‘negative’ of the pattern generated on the solid substrate. Figure 4*d* shows a gold mesh template coated with nHA and this is the inverse (negative) of the patterns. The HA nanoparticles distributed uniformly on the entire template and was of approximately 2 μm thickness as in the case of the substrate.

The biological response to nanoparticles of similarly electrosprayed HA coatings on titanium substrates has been investigated using simulated body fluid and is discussed elsewhere; in brief, bone-like apatite crystals formed *in vitro* (Li *et al*. in press).

## 4. Conclusions

We uncover and elucidate a new method for the creation of topography with bioactive materials. nHA topographies with different uniform micro-scale patterns have been successfully prepared on non-conducting glass and conducting titanium substrates by a TAEA spraying. The thickness of the coating is proportional to the spray time. This is an important forward step in regulating the topographies of bioactive substances on implant surfaces, particularly those of metallic origin.

## Figures and Tables

**Figure 1:**
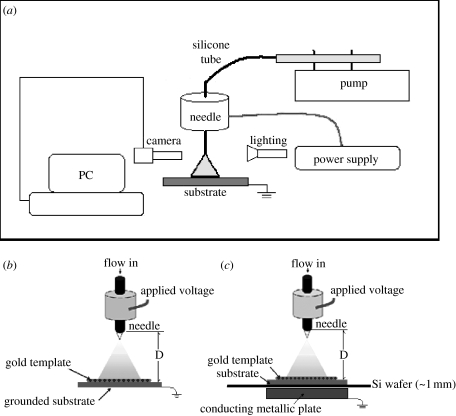
(*a*) Schematic illustrating the equipment set-up used to create nHA topography by generating electrohydrodynamic jetting with (*b*,*c*) showing the two different electrode configurations employed.

**Figure 2:**
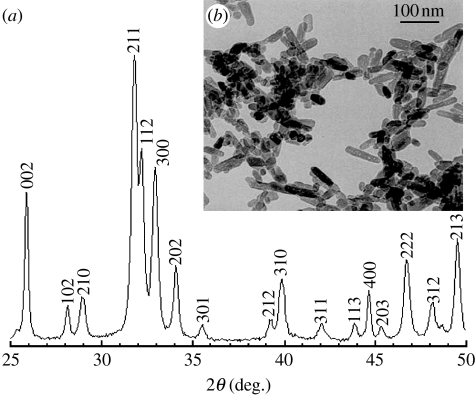
(*a*) X-ray diffraction pattern and (*b*) transmission electron micrograph of the nHA synthesized.

**Figure 3:**
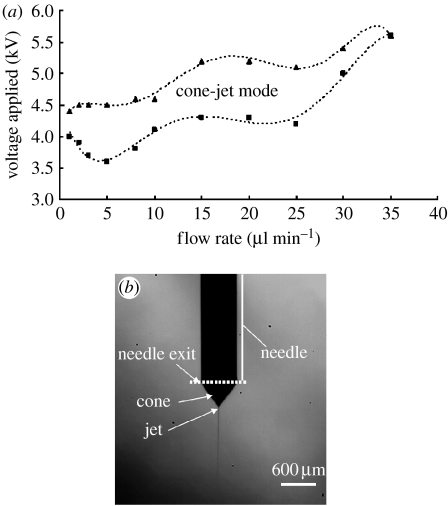
(*a*) Relationship between applied voltage and flow rate for the 6 wt% nHA suspension and (*b*) typical cone-jet mode obtained at 4.3–5.2 kV and 20 μl min^−1^.

**Figure 4:**
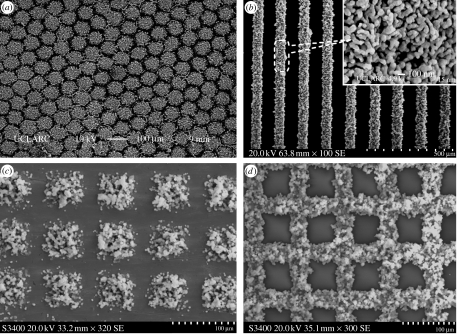
Scanning electron micrographs of (*a*) hexagonal nHA islands deposited on a glass substrate with the island diameter set at 50 μm, (*b*) nHA lines deposited on a titanium substrate with the width set at 50 μm, (*c*) square nHA islands deposited on a titanium substrate with the island diameter set at 50 μm and (*d*) nHA deposited on the gold template.
